# Vacancy Defects’ Effects on the Optoelectronic Properties of MAPbI_3_

**DOI:** 10.3390/ma18122694

**Published:** 2025-06-07

**Authors:** Wenchao Tang, Peiqi Ji, Ziyi Xu, Cuiping Xu, Jiaqi Dai, Xiyu Chen, Yawen Xu, Hongling Cai, Fengming Zhang, Xiaoshan Wu

**Affiliations:** 1 National Laboratory of Solid State Microstructures, Department of Physics, Nanjing University, Nanjing 210093, China; tangwenchao@smail.nju.edu.cn (W.T.); mg21220063@smail.nju.edu.cn (P.J.); mg21220121@smail.nju.edu.cn (Z.X.); 602023220075@smail.nju.edu.cn (C.X.); 602022220011@smail.nju.edu.cn (J.D.); cxiyu0615@163.com (X.C.); 652023220055@smail.nju.edu.cn (Y.X.); hlcai@nju.edu.cn (H.C.); fmzhang@nju.edu.cn (F.Z.); 2 Institute of Materials Engineering, Nantong 226019, China

**Keywords:** Perovskite MAPbI_3_, optoelectronic properties, point defects

## Abstract

This work derived the relationship between the concentrations of Pb^2+^ and I^−^ vacancies, along with MA^+^ vacancies, in MAPbI_3_ crystals and their effect on the short-circuit current of MAPbI_3_-based solar photovoltaic devices. First principles calculations revealed that Pb^2+^ and I^−^ vacancies introduce shallow defect levels near the Fermi level, acting as non-radiative recombination centers, which significantly influence the short-circuit current and open-circuit voltage. In contrast, MA^+^ vacancies have a negligible effect on the optoelectronic properties of MAPbI_3_. Based on this correlation, we successfully elucidated the declining trend of the short-circuit current (J_sc_) in MAPbI_3_-based devices with increasing Pb^2+^/I^−^ vacancy concentrations, while uncovering the microscopic mechanism responsible for the minor performance impact of MA^+^ vacancies.

## 1. Introduction

Perovskite solar cells (PSCs) utilizing organic–inorganic halide perovskites have recently attracted significant interest due to their exceptional photovoltaic performance. This high performance is caused by an ultra-high light absorption coefficient (approximately 10^5^ cm^−1^), a long carrier diffusion length (about 1 mm), and the production of free charges through the photoexcitation of organic–inorganic hybrid perovskite materials [1]. Notably, the bandgap of perovskite materials can be adjusted from 1.2 eV to 3.0 eV through compositional engineering [2,3,4]. This tunability has enabled the fabrication of perovskite/silicon tandem solar cells and perovskite/perovskite tandem solar cells which have theoretical efficiency limits exceeding 42 %, significantly surpassing the Shockley–Queisser limit for single-junction solar cells. The power conversion efficiency (PCE) of single-junction perovskite solar cells has rapidly advanced, with this type of solar cell achieving a certified PCE of 26.1% [5].

However, defects in perovskite materials, such as vacancies, interstitials, and antisite defects, significantly impact the performance of PSCs [6,7,8,9,10]. These defects form within the perovskite bandgap and can act as non-radiative recombination centers, thereby reducing the efficiency of the devices. The concentration of these defects is closely related to the performance of the battery devices. To enhance the efficiency and stability of PSCs, researchers are committed to studying and developing defect passivation strategies [11,12,13,14]. These strategies include chemical modification, ion doping, and interface engineering, all aimed at reducing the defect density, improving carrier transport, and minimizing non-radiative recombination [15,16,17]. Therefore, it is of paramount importance to conduct an in-depth study of these defects. Through theoretical simulation and calculation, we can reveal how these vacancy defects affect the material’s electronic structure and optoelectronic properties. Furthermore, different types and concentrations of vacancy defects have varying impacts on battery performance, with the determination of these providing theoretical guidance for experimental design and promoting the further optimization of perovskite solar cells. Existing studies have demonstrated that when the defect density at the CuI/CsPbI_3_ interface and in the photoactive layer exceeds 10^15^ cm^−3^, critical device performance parameters such as the open-circuit voltage (V_oc_) and fill factor undergo significant degradation [18]. Furthermore, the controlled modulation of shallow defect densities has been shown to enhance the V_oc_ from 1.05 V to 1.20 V, reaching 96.8% of the theoretical Shockley–Queisser (SQ) limit [19]. Investigations into defect formation energies in MAPbI_3_ have revealed that iodine vacancies (V_I_), lead vacancies (V_Pb_), and iodine interstitial defects (I_i_) constitute the dominant defect species that are detrimental to both material stability and optoelectronic performance [20]. These defects act as non-radiative recombination centers, accelerating carrier loss and impeding charge transport dynamics. The Shockley–Read–Hall (SRH) recombination model enables the quantitative calculation of carrier recombination rates using parameters such as the defect concentration, capture cross-sections, and carrier concentrations [21]. However, the SRH model necessitates the use of precise defect-specific parameters, which are often challenging to measure accurately in practice, particularly in complex material systems with multiple defect types or inhomogeneous distributions. Moreover, under high defect concentrations or non-equilibrium conditions, the model fails to adequately account for nonlinear interactions between carrier transport and defect dynamics, such as defect clustering, band renormalization, or tunneling-assisted recombination. These limitations may lead to reduced predictive accuracy in scenarios where defect-induced carrier localization or collective defect behavior dominates recombination pathways.

In this study, we first employed Density Functional Theory (DFT) to determine the electronic structure of Pb^2+^, MA^+^, and I^−^ vacancy defects in methylammonium lead triiodide (MAPbI_3_). By analyzing the energy level positions and density of states of these defect states, we elucidated the mechanisms of their influence on the material’s electronic properties. Furthermore, through our analysis, we derived the relationship between the vacancy concentrations and their impact on the short-circuit current and open-circuit voltage of MAPbI_3_-based solar photovoltaic devices. This relationship can serve as a guiding principle for subsequent enhancements in battery device performance. The model proposed in this study establishes a simplified parametric correlation between the defect concentration and macroscopic device performance metrics, such as the short-circuit current (J_sc_) and open-circuit voltage (V_oc_), thereby circumventing the necessity for an explicit dependence on intricate microscopic recombination mechanisms. This approach directly bridges defect-induced modifications in electronic structures and experimentally measurable performance parameters, eliminating the requirement for detailed inputs of defect energy levels or carrier capture cross-sections commonly mandated by conventional recombination models.

## 2. Calculation Methods

The computational investigations in this work were conducted employing a first principles Density Functional Theory (DFT) framework implemented using the Vienna Ab initio Simulation Package (VASP) code [22]. For the geometric optimization processes, the exchange–correlation interactions were treated using a Generalized Gradient Approximation (GGA) parameterized by the Perdew-Burke-Ernzerhof (PBE) functional [23]. To achieve enhanced electronic structure characterization, a dual-level computational strategy was adopted: the initial self-consistent field calculations were performed using a standard PBE functional, followed by high-accuracy hybrid functional computations employing the Heyd–Scuseria–Ernzerhof (HSE06) scheme for precise band structure analysis [24]. The electron–ion interactions were modeled using the Projector-Augmented Wave (PAW) pseudopotential approach with an optimized plane wave energy cutoff of 400 eV to ensure the convergence of the total energy calculations. Structural relaxations were executed using conjugate gradient optimization algorithms with stringent convergence criteria—the total energy tolerance was set at 1 × 10^−5^ eV, and the maximum atomic force components were constrained below 0.02 eV/Å. The computational protocol involved the sequential optimization of both the lattice parameters and atomic coordinates prior to subsequent electronic structure analyses. Reciprocal space sampling was implemented using Γ-centered Monkhorst–Pack grids with a k-point spacing density controlled at 0.02 × 2π/Å for Brillouin zone integrations. To enhance the computational efficiency and workflow management, the vaspkit toolkit (version 1.3.1) was systematically employed for input file generation and post-processing operations [25].

The defect formation energy was calculated using the following formula:

(1) $$∆E_{f}=E_{defect}-E_{perfect}-\sum n_{i}\mu_{i}+q\left(E_{VBM}+E_{F}+∆V\right)+E_{corr}$$

where $E_{defect}$ denotes the total energy of the supercell containing the defect, $E_{perfect}$ represents the total energy of the pristine (defect-free) supercell, $n_{i}$ indicates the change in the number of atoms/ions within the supercell due to defect formation, $\mu_{i}$ corresponds to the chemical potential of the respective atom/ion, q refers to the charge state of the defect, $E_{VBM}$ signifies the energy of the valence band maximum, used to correlate the charge state with the electron chemical potential, $E_{F}$ denotes the Fermi level of the system, ∆V represents the electrostatic potential correction term to mitigate errors induced by periodic boundary conditions in supercell calculations, and $E_{corr}$ accounts for additional correction terms (e.g., finite-size effects or image charge corrections) [26,27,28]. The specific correction scheme employed in this work was the Makov–Payne (MP) correction [29], expressed as

(2) $$E_{corr}=\frac{q^{2}\alpha_{M}}{2\varepsilon L}+\frac{2\pi qQ}{3\varepsilon L^{3}}$$

where $\alpha_{M}$ denotes the Madelung constant for the finite supercell geometry, ε represents the static dielectric constant of the material, and L corresponds to the characteristic dimension of the supercell.

## 3. Results and Discussion

**Figure 1:**
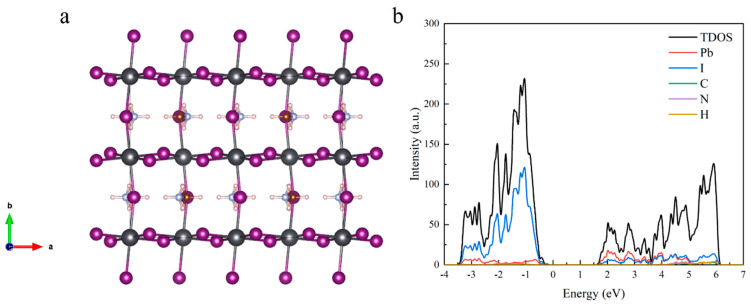
(**a**) The structure and (**b**) partial density of state of MAPbI_3_ after structural optimization. The gray spheres represent Pb atoms; the purple spheres represent I atoms; and the structure between Pb and I represents the MA^+^ cations.

We constructed a 2 × 1 × 2 supercell of MAPbI_3_ to determine the electronic structure of both the ideal crystal and the defective crystal. Figure 1 shows the cell structure of MAPbI_3_ without defects and the partial density of states (DOS) of the electrons. The optimized MAPbI_3_ unit cell crystallized in the I4/mcm space group, comprising four primitive unit cells of MAPbI_3_ with lattice parameters of a = c = 8.695 Å and b = 12.834 Å. The Pb–I bond length was measured as 3.18 Å, with an I–Pb–I bond angle of 169.3°. For comparison, the experimental lattice parameters of the tetragonal-phase MPI were a = 8.85 Å and b = 12.634 Å.

Defect-related calculations were performed by constructing supercells through the addition or removal of defect atoms/ions at high-symmetry positions, as shown in Figure 1a. Positioning the defects at these symmetry-constrained sites minimized the structural distortions caused by arbitrary defect placement, thereby enhancing the computational reliability and reducing artifacts.

We first determined the interstitial defects induced by several atomic species. Specifically, the interstitial defects caused by carbon (C) and nitrogen (N) generated deep-level defects near the Fermi level. These deep-level defects exhibited significantly stronger carrier trapping capabilities compared to shallow-level defects. Electrons captured by such defects were less likely to be re-excited into the conduction band, thereby exerting a more pronounced influence on the carrier dynamics. This work primarily focused on establishing a semi-quantitative formulation (Equation (12)) based on the vacancy defects in MAPbI_3_. Although the explicit mechanisms of interstitial defects were not independently modeled, their collective effects were encapsulated through the use of the parameters m (non-radiative recombination coefficient) and p (defect compensation factor). This parametrization framework effectively generalized the contributions of diverse interstitial types to the device performance while preserving the model’s simplicity and predictive capability. Consequently, the subsequent analysis will primarily focus on the impact of vacancy defects on the electronic structure of MAPbI_3_.

For the cases containing different vacancy defects, our calculation results were as follows.

**Figure 2:**
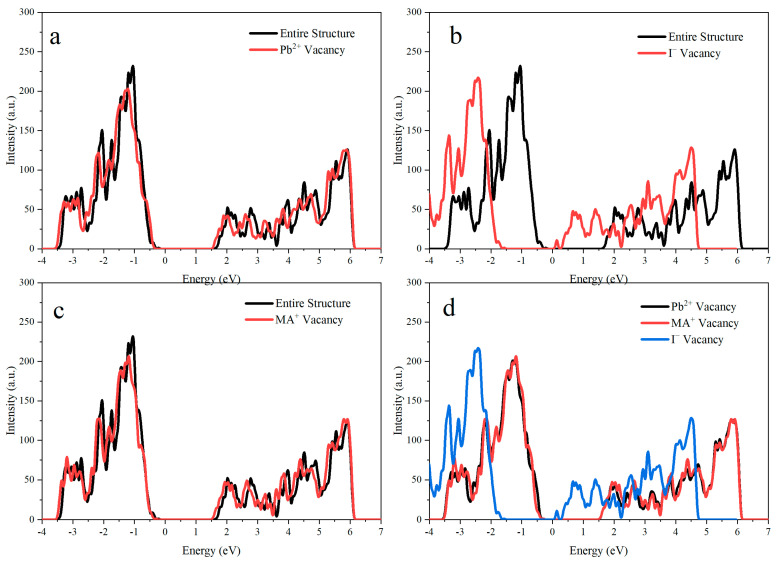
(**a**) Comparison of the electronic density of states (DOS) for Pb^2+^ vacancies in MAPbI_3_ versus the defect-free structure, (**b**) I^−^ vacancies in MAPbI_3_ versus the defect-free structure, and (**c**) MA^+^ vacancies in MAPbI_3_ versus the defect-free structure, and (**d**) a comparative analysis of the DOS for all three types of point defects in MAPbI_3_.

Figure 2a illustrates the comparison between the total density of states (TDOS) of the entire structure (black curve) and that of the Pb^2+^ vacancy (red curve). The complete structure exhibited a low density of states near the Fermi level at zero, signifying a distinct bandgap. The presence of Pb^2+^ vacancies notably enhanced the density of states near the valence band’s upper region. These states were located within the bandgap but closer to the valence band edge, suggesting that they were likely shallow defect states. Figure 2b compares the complete structure and the I^−^ vacancy. Like Figure 2a, the full structure exhibits a low density of states near the Fermi level; however, the presence of an I^−^ vacancy adds extra density of states at the Fermi level. The density of states decreases at both the conduction band’s lower edge and the valence band’s upper edge. Figure 2c compares the TDOS of the complete structure and the MA^+^ vacancy. The MA^+^ vacancy contributed to the appearance of additional states near the top of the valence band, though its impact on the density of states was less significant than that of the Pb^2+^ or I^−^ vacancies. This suggests that MA^+^ vacancies introduce weaker shallow defect states. Finally, Figure 2d compares all three types of vacancies: Pb^2+^, MA^+^, and I^−^. From this figure, it is evident that all three vacancy types introduced an additional density of states, but their magnitudes and distributions differed. The Pb^2+^ and I^−^ vacancies significantly increased the density of states, whereas the MA^+^ vacancy had a comparatively minor impact.

**Figure 3:**
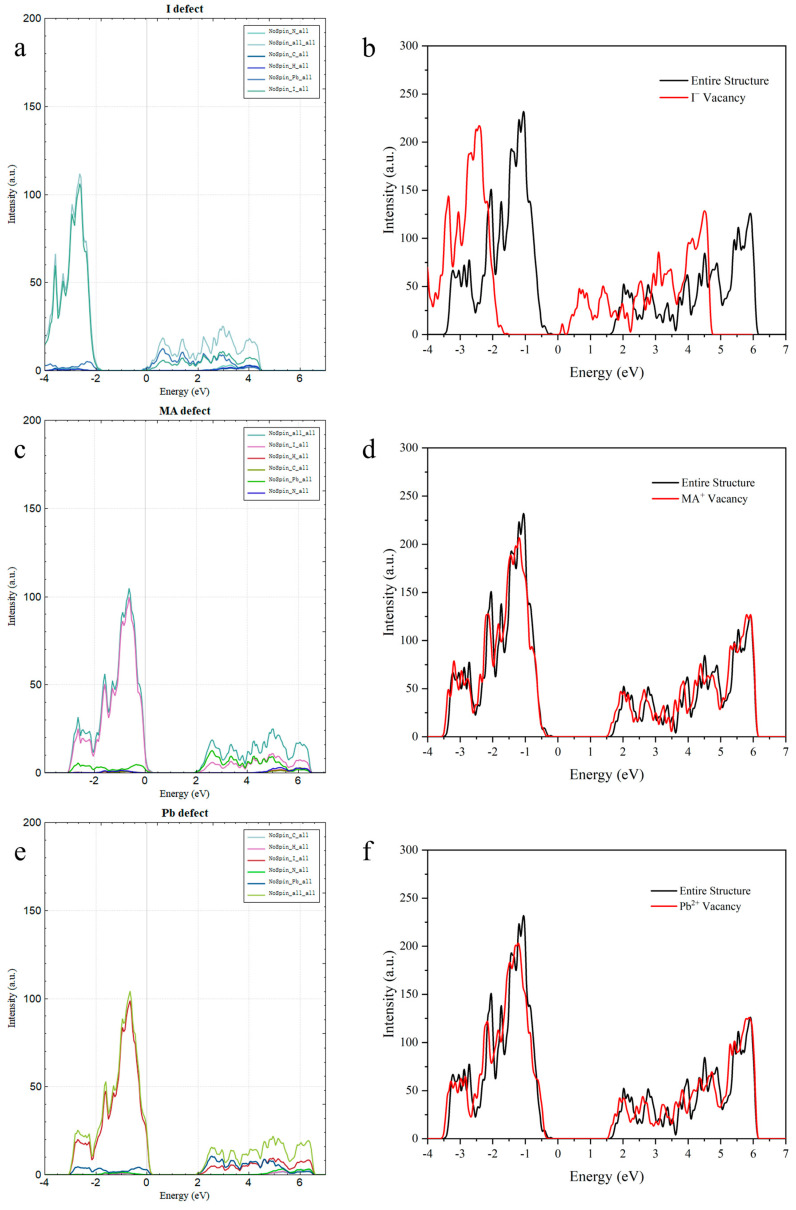
Comparative analysis of the I^−^ defects (**a**,**b**), MA^2+^ defects (**c**,**d**), and Pb^2+^ defects (**e**,**f**) computed using the DS-PAW (**a**,**c**,**e**) and VASP (**b**,**d**,**f**) methodologies.

To further verify the rationality and reliability of our calculations, we also employed DS-paw within Device Studio to recalculate and validate the defective structures, as shown in Figure 3, yielding nearly consistent results. The minor numerical differences observed were primarily attributable to inconsistencies in the computational parameters across different software platforms.

**Figure 4:**
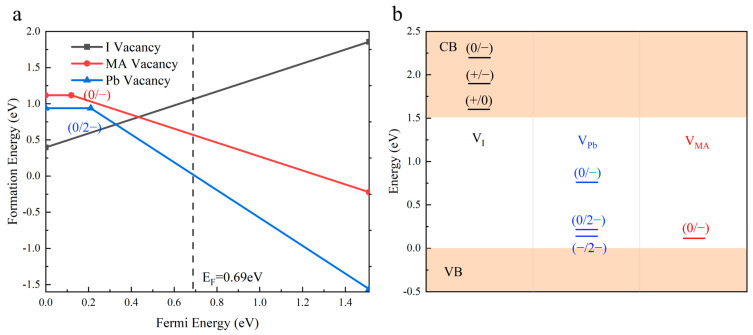
(**a**) Formation energies of vacancy defects in MAPbI_3_ as function of chemical potential; (**b**) transition levels of defects.

Furthermore, under selected moderate iodine chemical potential conditions, we systematically calculated the formation energies and transition levels of various vacancy-type defects in MAPbI_3_, with the computational results comprehensively summarized in Figure 4.

As illustrated in Figure 4, the stable charge states of the iodine vacancies (V_I_) in MAPbI_3_ resided entirely within the conduction band, indicating their inability to form deep-level defects. For lead vacancies (V_Pb_), three charge states (−/2−) and (0/−) exhibited excessively high formation energies, rendering them thermodynamically unfavorable. The only viable charge state (0/2−) suggested that V_Pb_ tends to capture holes near the valence band maximum (VBM), characteristic of shallow-level defect behavior. Similar shallow-level characteristics were observed for methylammonium vacancies (V_MA_) in their (0/−) charge state. Notably, under iodine-rich conditions (high μ_I_), the formation energy of V_I_ increased while that of V_Pb_ decreased, promoting the preferential formation of V_Pb_ defects. Conversely, iodine-deficient conditions (low μ_I_) favored V_I_ defect formation, demonstrating an inverse energy trend.

Some studies have noted that by employing MoSe_2_ to control the film formation rate of MAPbI_3_, the optoelectronic performance of batteries can be significantly improved [30,31]. Such regulation effectively reduces the presence of various defects in MAPbI_3_, leading to an increase in the short-circuit current and photovoltaic conversion efficiency of battery devices. Based on the preceding analysis, vacancy defects represented by Pb^2+^, MA^+^, and I^−^, especially those associated with Pb^2+^ and I^−^, have a marked influence on the optoelectronic properties of perovskite materials. To further comprehend how these point defects affect battery devices, we conducted a semi-quantitative analysis of the material’s optoelectronic properties, particularly the impact on the short-circuit current, by calculating the changes in the effective masses of electrons and holes due to these defects, focusing solely on the perovskite layer within these devices.

First, according to our calculations, the electron effective mass of a MAPbI_3_ unit cell without any defects is 0.152m_0_, and the hole effective mass is 0.137m_0_, where m_0_ is the rest mass of the electron. Initially, for a MAPbI_3_ structure with N unit cells, the defect concentration (η) is defined as follows [19,32]:

(3) $$\eta =\frac{n_{number\;of\;defects}}{N}\times 100\%$$

Anion defects primarily affect the recombination of electrons, while cation vacancy defects mainly affect the recombination of holes. Therefore, for vacancy defects of the corresponding concentrations, we have calculated the effective masses of electrons and holes, respectively. The effective mass of electrons in a MAPbI_3_ unit cell containing one I^−^ vacancy is 0.252m_0_, the effective mass of holes with one MA^+^ vacancy is 0.15m_0_, and the effective mass of holes with one Pb^2+^ vacancy is 0.55m_0_. It can be observed that defects increase the effective mass of electrons, which implies a decrease in the carrier mobility. Furthermore, we have derived the relationship between the effective mass and short-circuit current.

Firstly, the impact of vacancy defects on the effective mass of perovskites is as follows: vacancy defects alter the band structure of perovskite materials, thereby affecting their effective mass. The variation in the effective mass can be reflected by the following formula [33]:

(4) $$m^{*}=\frac{\hbar^{2}}{k\left(\frac{d^{2}E}{dk^{2}}\right)}$$

in which the variation in $\frac{d^{2}E}{dk^{2}}$ reflects the changes in the band curvature caused by vacancy defects. Therefore, the vacancy concentration $\eta_{v}$ affects $m^{*}$ by altering $\frac{d^{2}E}{dk^{2}}$. If vacancies introduce localized states, they may reduce $\frac{d^{2}E}{dk^{2}}$, thereby leading to an increase in $m^{*}$.

To more accurately describe this relationship, we assume that there are n different types of vacancy defects; then, the change in the effective mass is proportional to the concentration of vacancy defects:

(5) $$m^{*}\left(\eta_{v_{1}},\eta_{v_{2}},\ldots ,\eta_{v_{n}}\right)=m_{0}^{*}\left(1+\sum_{i}\alpha_{i}\eta_{v_{i}}\right)$$

in which $m_{0}^{*}$ is the effective mass in the absence of any vacancy defects, and $\alpha_{i}$ is the constant that represents the impact of a specific vacancy defect, $v_{i}$, on the effective mass.

Next, we continue to analyze the relationship between the effective mass and the short-circuit current in perovskite solar cells. First, the effective mass is closely related to the mobility of charge carriers, which in turn affects their ability to move in an electric field.

Specifically, the relationship between the mobility (*μ*) and effective mass can be expressed as

(6) $$\mu =\frac{q\tau }{m^{*}}$$

where *e* is the elementary charge, and *τ* is the carrier mean scattering time, which corresponds to the average time interval between successive scattering events [34].

In solar cells, the drift velocity and diffusion velocity of charge carriers both contribute to the total current. Considering the drift–diffusion equation, the short-circuit current has the following relationship:

(7) $$J_{sc}=q\mu nE+qD_{n}\nabla n$$

where *E* represents the electric field strength, *q* represents the charge, $D_{n}$ is the diffusion coefficient of the charge carriers, and *n* represents the concentration of charge carriers. Under short-circuit conditions, the electric field strength is zero, and the relationship between the diffusion coefficient and mobility is as follows [35]:

(8) $$D_{n}=\mu \frac{kT}{q}$$

Substituting the previous equations, we get

(9) $$J_{sc}=\mu kT\nabla n=\frac{q\tau }{m^{*}}kT\nabla n=\frac{q\tau }{\sqrt{m_{e0}^{*}\left(1+\sum_{i}\alpha_{i}\eta_{v_{i}}\right)m_{h0}^{*}\left(1+\sum_{j}\alpha_{j}\eta_{v_{j}}\right)}}kT\nabla n$$

where *k* represents the Boltzmann constant, and *T* represents the temperature. Of course, we also need to continue considering the relationship between the concentration of charge carriers and the concentration of vacancy defects. First, the concentration of charge carriers (*n*) is usually determined by the doping concentration and intrinsic carrier generation. Vacancy defects may affect the concentration of charge carriers by providing or removing charge carriers, depending on their position in the band structure.

Owing to the assumption of the homogeneous distribution of vacancy defects, the concentration gradient’s contribution to carrier transport can be neglected; therefore, the short-circuit current is primarily dominated by the effective mass variation. We assume that the concentration gradient of vacancies is approximately zero, so

(10) $$\nabla n=\nabla n_{0}-\nabla \eta_{v_{i}}f\left(E\right)\approx \nabla n_{0}$$

Thus, the short-circuit current should now be

(11) $$J_{sc}=\frac{1}{\sqrt{\left(1+\sum_{i}\alpha_{i}\eta_{v_{i}}\right)\left(1+\sum_{j}\alpha_{j}\eta_{v_{j}}\right)}}\frac{q\tau kT\nabla n_{0}}{m_{0}^{*}}$$

Based on the existing data, we have calculated

$$\alpha_{I}=7.895\;\alpha_{Pb}=12.058\;\alpha_{MA}=0.379$$

Comparing the impact constants, the influence of MA vacancies on the effective mass is much smaller compared to that of Pb and I vacancies, which is consistent with the conclusions drawn from the electron density of states calculations mentioned earlier. Therefore, in MAPbI_3_, if we only consider the changes in these three types of vacancy defects, the relationship between the short-circuit current and the three vacancy defects is

(12) $$J_{sc}=\frac{1}{\sqrt{\left(1+7.895\eta_{I}+m\right)\left(1+12.058\eta_{Pb}+0.379\eta_{MA}+p\right)}}\frac{q\tau kT\nabla n_{0}}{m_{0}^{*}}$$

where *m* and *p* represent the impact of other defects on the effective masses of electrons and vacancies, respectively.

According to the experimental data from the literature, the average free time τ of charge carriers is in the range of roughly $10^{-14}\sim 10^{-13}$ seconds, and the gradient of the charge carrier concentration is between $10^{16}\;and\;10^{19}\;{cm}^{-4}$ [36,37,38,39]. The concentration of charge carriers and the average free time *τ* vary greatly among different devices due to environmental factors, such as the light intensity, humidity, etc., making it difficult to accurately estimate *τ* and ∇*n*. Therefore, in this calculation, we take the average values from the range, so

$$\frac{q\tau kT\nabla n_{0}}{m_{0}^{*}}\approx 57.32\;mA/{cm}^{2}$$

Firstly, according to our calculations, when there are no defects in the cell,

$$J_{sc}=57.32\;mA/{cm}^{2}$$

This value is slightly higher than the typical range of short-circuit currents for perovskite cells, but it is considered reasonable since it is an ideal case.

For general cases, the defect density is between approximately 10^16^ and 10^17^ cm^−3^. When converted to the concentration, it should be roughly 0.1% to 0.3%. Considering *m* = *p* = 1.5, we have

$$J_{sc0}=22.65\;mA/{cm}^{2}$$

After optimizing the defects in the cell by controlling the film formation rate of MAPbI_3_, we believe that the concentration of various defects should be uniformly reduced. Assuming that the defect concentration is reduced from 0.3% to 0.25%,

$$J_{sc5}=25.19\;mA/{cm}^{2}$$

The calculated values are basically consistent with the experimental results in the literature, and the trend of change is also additive. The reason for the calculated values being higher than the experimental values is that other factors affecting the short-circuit current were not considered, such as the impact of hole transport layers, electron transport layers, interlayer defects, etc., so these values are reasonable.

In addition, we investigated the semi-quantitative relationship between the effective mass of perovskite materials and the open-circuit voltage ($V_{oc}$) of devices. It was noted that the carrier effective mass directly influences the density of states (DOS) and intrinsic carrier concentration ($n_{i}$) of the material, thereby affecting the $V_{oc}$.

Specifically, the DOS in the conduction band ($N_{c}$) and valence band ($N_{v}$) exhibits a functional dependence on the effective mass, which can be expressed as

(13) $$N_{c}=2{\left(\frac{2\pi m_{e}^{*}kT}{h^{2}}\right)}^{\frac{3}{2}},N_{v}=2{\left(\frac{2\pi m_{h}^{*}kT}{h^{2}}\right)}^{\frac{3}{2}}$$

The intrinsic carrier concentration is represented as

(14) $$n_{i}=\sqrt{N_{c}N_{v}}e^{-\frac{E_{g}}{2kT}}\propto {\left(m_{e}^{*}m_{h}^{*}\right)}^{\frac{3}{4}}e^{-\frac{E_{g}}{2kT}}$$

In the open-circuit voltage ($V_{oc}$) model governed by non-radiative recombination, $V_{oc}$ can be expressed as [40,41]

(15) $$V_{oc}\approx \frac{E_{g}}{q}-\frac{kT}{q}\ln\left(\frac{\eta \sigma \upsilon_{th}n_{i}}{G}\right)\propto \ln\left(\frac{1}{\eta {\left(m_{e}^{*}m_{h}^{*}\right)}^{\frac{3}{4}}}\right)$$

In this context, G represents the photogenerated carrier generation rate, $\upsilon_{th}$ denotes the carrier thermal velocity, and σ signifies the defect capture cross-section. An increase in the point defect concentration (η) results in higher effective masses for both electrons and holes. An enhancement in the defect concentration (η) simultaneously amplifies the effective masses of both types of charge carriers (electrons and holes), thereby reducing the open-circuit voltage ($V_{oc}$) via the non-radiative recombination mechanism described by Equation (15). This trend exhibits good agreement with relevant experimental data, underscoring the critical role of defect-mediated non-radiative recombination in determining the limitations of perovskite-based photovoltaic systems.

## 4. Conclusions

In this study, we thoroughly investigated the impact of different vacancy defects on the optoelectronic performance of MAPbI_3_ perovskite solar cells. Through calculations using Density Functional Theory (DFT), we found that Pb^2+^, I^−^, and MA^+^ vacancies introduce an additional density of states near the Fermi level, indicating that they introduce shallow-level defects. These defects may reduce the optoelectronic performance of the material by increasing the recombination centers in the bandgap. The increase in the density of states introduced by Pb^2+^ and I^−^ vacancies is more significant and may have a greater negative impact on performance, while the impact of MA^+^ vacancies is relatively smaller. Furthermore, we found that these defects increase the effective masses of electrons and holes, thereby reducing the carrier mobility and affecting the short-circuit current and open-circuit voltage by altering the effective mass. Ultimately, through validations and comparisons with the existing literature, we determined that our research results are consistent with experimental data, proving that reducing these defects can effectively enhance the optoelectronic performance of solar cells. The present study focused on the influence of bulk defects on device performance, while the interaction between interface/surface defects was not incorporated in the current model. This omission constitutes a notable limitation of the present framework, which will be addressed in subsequent investigations through the integration of interface engineering to systematically analyze the synergistic effects of surface and interfacial defects. In summary, this study not only reveals the specific impact of different vacancy defects on the optoelectronic performance of MAPbI_3_ perovskite solar cells but also provides a theoretical basis for optimizing the performance of perovskite solar cells through defect engineering.

## Data Availability

The data that supports the findings of this study are available within the article.
